# Kinetics of Wound Development and Healing Suggests a Skin-Stabilizing Effect of Allogeneic ABCB5^+^ Mesenchymal Stromal Cell Treatment in Recessive Dystrophic Epidermolysis Bullosa

**DOI:** 10.3390/cells12111468

**Published:** 2023-05-24

**Authors:** Elke Niebergall-Roth, Kathrin Dieter, Cristina Daniele, Silvia Fluhr, Maria Khokhrina, Ines Silva, Christoph Ganss, Markus H. Frank, Mark A. Kluth

**Affiliations:** 1 RHEACELL GmbH & Co. KG, 69120 Heidelberg, Germany; 2 Department of Dermatology, Brigham and Women’s Hospital, Harvard Medical School, Boston, MA 02115, USA; 3 Harvard Stem Cell Institute, Harvard University, Cambridge, MA 02138, USA; 4 Transplant Research Program, Boston Children’s Hospital, Harvard Medical School, Boston, MA 02115, USA; 5 School of Medical and Health Sciences, Edith Cowan University, Perth 6027, Australia

**Keywords:** ABCB5, cell therapy, mesenchymal stromal cells, recessive dystrophic epidermolysis bullosa, wound healing

## Abstract

Recessive dystrophic epidermolysis (RDEB) is a rare, inherited, and currently incurable skin blistering disorder characterized by cyclically recurring wounds coexisting with chronic non-healing wounds. In a recent clinical trial, three intravenous infusions of skin-derived ABCB5^+^ mesenchymal stromal cells (MSCs) to 14 patients with RDEB improved the healing of wounds that were present at baseline. Since in RDEB even minor mechanical forces perpetually provoke the development of new or recurrent wounds, a post-hoc analysis of patient photographs was performed to specifically assess the effects of ABCB5^+^ MSCs on new or recurrent wounds by evaluating 174 wounds that occurred after baseline. During 12 weeks of systemic treatment with ABCB5^+^ MSCs, the number of newly occurring wounds declined. When compared to the previously reported healing responses of the wounds present at baseline, the newly occurring wounds healed faster, and a greater portion of healed wounds remained stably closed. These data suggest a previously undescribed skin-stabilizing effect of treatment with ABCB5^+^ MSCs and support repeated dosing of ABCB5^+^ MSCs in RDEB to continuously slow the wound development and accelerate the healing of new or recurrent wounds before they become infected or progress to a chronic, difficult-to-heal stage.

## 1. Introduction

Recessive dystrophic epidermolysis bullosa (RDEB) is a rare inherited skin blistering disorder in which total loss or deficiency in functional collagen VII at the dermo–epithelial junction causes excessive skin fragility and progressive multi-organ fibrosis [1,2,3]. Effective systemic curative therapies targeting this underlying genetic defect are not available for routine clinical care so far [4,5], pointing to an urgent need for disease-modifying treatments that effectively improve defective wound healing and alleviate severe symptoms such as itch and pain [6]. In addition to the investigation of several small-molecule-based drug therapy approaches [7], the association of RDEB with systemic inflammation beyond skin-limited involvement [2,3,8] has stimulated the development of cell-based therapeutic approaches including allo-transplantation of mesenchymal stromal cells (MSCs) such as human bone marrow- and umbilical cord blood (UCB)-derived MSCs [9,10,11]. Recently, a skin-resident immunomodulatory MSC population, characterized by expression of the ABC transporter ABCB5 [12,13], has facilitated the healing of acute and chronic skin wounds after topical administration in preclinical and clinical studies [13,14,15,16,17]. In addition, following systemic intravenous grafting, ABCB5^+^ MSCs reduced RDEB symptoms and significantly prolonged the lifespan in a *Col7a1*^–/–^ mouse model of RDEB [18]. Very recently we found that three intravenous infusions of allogeneic skin-derived ABCB5^+^ MSCs to patients with RDEB decreased disease activity, alleviated itch and pain, and facilitated the healing of the wounds that were present at baseline [19,20].

However, unless a treatment was capable of not only healing existing wounds but also of restoring deficient collagen VII expression at the dermo-epithelial junction, even minor mechanical forces would continue to provoke new wound development. Consequently, in the absence of causal RDEB cures, there exists an urgent need for maintenance therapies capable of slowing new wound development and/or accelerating new wound healing before the onset of possible wound infection or of wound progression to chronic, more difficult-to-heal stages. Given the pronounced anti-inflammatory and ECM-remodeling effects that have been attributed to ABCB5^+^ MSCs in various preclinical and clinical settings [21], we wondered whether these cells might be a candidate for such a preventative wound reduction maintenance treatment approach. To this end, we conducted an exploratory post-hoc analysis of the above-referenced clinical trial in RDEB patients, specifically focusing on the development and healing kinetics of wounds that were not present at baseline during 12 weeks of systemic treatment with ABCB5^+^ MSCs.

## 2. Materials and Methods

### 2.1. Clinical Trial

The clinical trial design, inclusion and exclusion criteria, and the results for all pre-defined outcome measures have been reported previously [19]. To summarize, 16 adult and pediatric patients with genotypically and phenotypically diagnosed RDEB were enrolled at five study sites in Germany, Austria, France, the United Kingdom, and the United States, to receive three intravenous infusions of 2 × 10^6^ allogeneic ABCB5^+^ MSCs/kg, manufactured as a standardized GMP-compliant advanced-therapy medicinal product [22,23] (for product release data see [19]), on days 0, 17, and 35. The patients were followed up for 12 weeks regarding the efficacy and for one year regarding safety.

The trial was conducted according to the guidelines of the Declaration of Helsinki. The protocol and all other relevant documents had been approved by the relevant drug regulatory authorities and the local independent ethics committees/institutional review boards. Prior to any trial-related procedures, written informed consent was obtained from all patients or, in the case of minors, their parents.

### 2.2. Photograph Assessments

**Figure 1:**
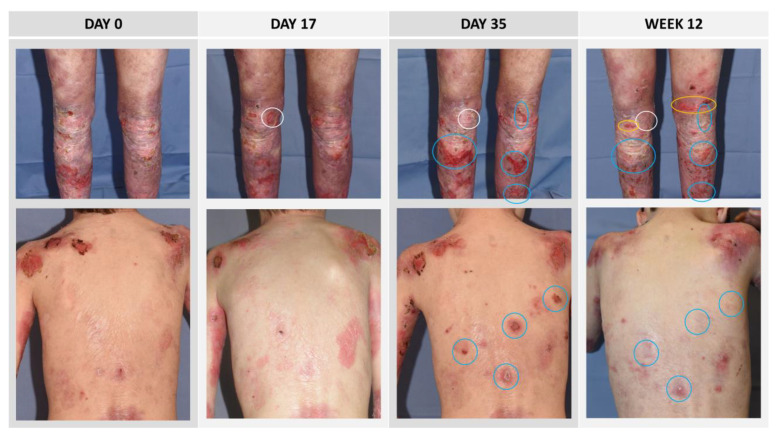
Sample series of patient photographs. New wounds, defined as wounds that were not present at baseline (day 0), were marked on the day they first occurred, and the wound areas were followed up to week 12. Wounds that first occurred on day 17 are circled in white, wounds that first occurred on day 35 are circled in blue, and wounds that first occurred on week 12 are circled in orange. Upper panel: Female patient, 36 years. Lower panel: Male patient, 9 years. The patients or their parents consented to the publication of their photographs.

At each efficacy visit (day 0, day 17, day 35, week 12), photographs of the affected body regions were taken for documentary purposes. In situations where this would have imposed undue stress on the patient, the investigator was allowed to desist from photographing the respective body area(s) at any visit. In the present post-hoc analysis, all wounds in all body regions of which photographs were taken at all four efficacy visits were included. The photographs were independently assessed by three reviewers to record the number of new wounds, defined as wounds that were open at any post-baseline visit but had not been open at day 0. Exemplary series of evaluated photographs are shown in Figure 1.

### 2.3. Calculations

The numbers of the observed new wounds were summed up over all patients, and grouped according to the time point of their first observation (designated as “day-17”, “day-35” and “week-12 new wounds”). For the day-17 and day-35 new wounds, the following outcome parameters were calculated, as applicable: healing ratio, defined as the percentage of wounds that had healed until each of the subsequent visits; median time to wound closure; and proportion of durably healed wounds, defined as the percentage of closed wounds that remained closed over a period that exceeded the typical time to recurrence, determined in natural-history studies on RDEB as about 3 weeks on average [24] (in the present analysis at least 7 weeks). Where possible, the results were compared to the corresponding, previously published results for the wounds that were present at baseline [20]. Descriptive statistics were employed to summarize the data of this post-hoc analysis.

## 3. Results

### 3.1. Incidence of New Wounds

**Figure 2:**
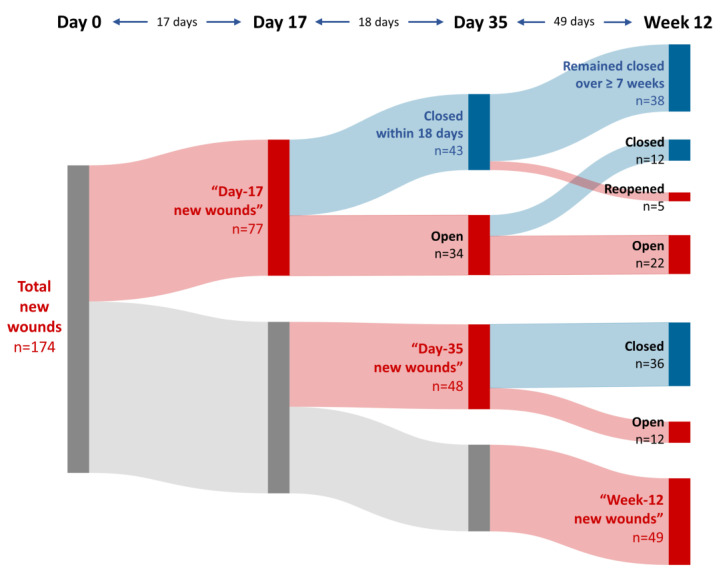
Sankey diagram indicating the occurrence and outcome of 174 wounds that had not been present at baseline (“new wounds”) in 14 RDEB patients during treatment with ABCB5^+^ MSCs. For each time point, red nodes represent open wounds and blue nodes represent healed wounds.

Photograph series covering all four efficacy visits were available from 14 patients (6 male, 8 female, age 6–36 years). In total, 174 wounds that were not present at the baseline visit could be followed up. Of these, 77 wounds (44%) developed over the first 17 days (“day-17 new wounds”), whereas only 48 wounds (28%) occurred over a further 18 days (until day 35, “day-35 new wounds”). The remaining 49 wounds (again 28%) were observed only at week 12 (“week-12 new wounds”), which means that they developed over a period (from day 35 till week 12) that was almost three times as long (49 days as compared to 17 days for the day-17 wounds and 18 days for the day-35 wounds) (Figure 2).

### 3.2. Outcomes of the New Wounds

Of the 77 wounds that occurred for the first time on day 17 (“day-17 new wounds”), 43 wounds (56%) had already closed again by day 35. This means that more than half of these wounds had healed within a maximum of 18 days (the period between day 17 and day 35), which translates into a median time to first wound closure of 18 days. Remarkably, a very large portion (88%, 38 wounds) out of the 43 wounds that had closed again already by day 35 was still closed at the week-12 visit, i.e., remained closed over at least 7 weeks. A further 12 wounds of the day-17 new wounds closed between day 35 and week 12, so that in total approximately two-thirds (50 out of 77) of day-17 new wounds had closed by week 12 (Figure 2).

The 48 wounds that occurred for the first time on day 35 (“day-35 new wounds”) were followed up only at one subsequent visit, i.e., at week 12. Of these wounds, three-quarters (36 wounds, 75%) were closed at week 12, i.e., had healed within 7 weeks or less (Figure 2).

### 3.3. Comparison of New versus Baseline Wounds

The outcomes of the new wounds were compared with those of the baseline wounds previously reported in the same patient population [20], taking into account that the different groups of wounds, depending on the time point at which they were detected, were monitored over time intervals with different lengths (baseline wounds, 12 weeks; day-17 new wounds, 9.5 weeks; day-35 new wounds, 7 weeks) (Table 1). The comparison showed that more than twice the proportion of day-17 new wounds (56%) as compared to the baseline wounds (27%) healed in the short time (18 days and 17 days, respectively; Table 1). In line, the median time to first wound closure for the day-17 new wounds (18 days) was only half that of the baseline wounds (35 days). Furthermore, follow-up of these rapidly healing day-17 new wounds revealed that a greater portion of (i.e., 88%) remained closed over at least 7 weeks after closure compared to the 74% proportion of the baseline wounds (Table 1).

## 4. Discussion

Even though RDEB has been recognized to display features of systemic inflammation leading to progressive multi-organ fibrosis [2,3,8], skin wound closure is consistently identified among the most desired outcomes of disease-modifying treatment [25,26]. As recently reported, systemically administered allogeneic ABCB5^+^ MSCs have emerged as capable of facilitating complete and durable wound closure in patients with RDEB [20]. However, in view of the complex and highly dynamic RDEB skin wound evolution composed of chronic persistent and recurrent healing/re-opening wounds [24,27], an ideal wound closure strategy would not only target already existing wounds but also induce slowing of the occurrence of newly developing or recurring wounds and/or facilitate and accelerate healing once such wounds have developed before they enlarge, become infected or become chronic.

An indication that treatment with ABCB5^+^ MSCs might indeed have been capable of delaying the occurrence of new (i.e., not present at baseline) wounds is provided by the distribution of the newly occurred wounds over the different time points of observation: After nearly half of these wounds (44%) had occurred already by day 17, only 28% of wounds occurred after another 18 days (on day 35), whereas another 28% of wounds developed only over a comparatively long period of a further 7-week time interval (until week 12) (Figure 2).

Moreover, when compared to the previously reported healing responses of the baseline wounds to treatment with ABCB5^+^ MSCs [20], the new wounds displayed improved outcomes. Most strikingly, day-17 wounds showed an approximately two-fold proportion of rapidly healing wounds (56% within 18 days) compared to baseline wounds (27% within 17 days), which corresponded to approximately half the median time to first wound closure (18 days versus 35 days for baseline wounds) (Table 1). In addition, a greater proportion (88%) of these early-healing new wounds, compared to 74% of early-healing baseline wounds [20], remained stably closed over at least 7 weeks (Figure 2), i.e., they remained closed at least two-fold longer than the typical average time observed for closed wounds to reopen in RDEB (3 weeks) [24]. For the day-35 wounds, a direct comparison of the wound healing parameters with those of the baseline wounds was impeded by the different lengths of the follow-up periods (see Table 1), owing to the varying intervals between the trial visits. Nevertheless, while 65% of the baseline wounds were closed at the end of the 12-week treatment and efficacy phase, 75% of the day-35 wounds were closed after just over half the time (7 weeks) (Table 1). This might indicate an earlier treatment response also in the day-35 wounds compared to baseline wounds.

It is important to note that by specifically monitoring the wounds that developed during the treatment period, the present analysis was able to distinguish between the two types of wounds, i.e., chronic persistent and recurrent healing/re-opening wounds, which typically co-exist in RDEB patients [24,27]. While the baseline wounds included both types of wounds, including a significant proportion of chronic wounds, the newly developed wounds displayed exclusively the non-chronic, recurrent wound type. The herein observed faster and greater healing successes of new wounds compared to baseline wounds might be explained by the fact that with repeated cell dosing, successive MSCs delivered after the first dosing had homed to new wounds at earlier time points following their first occurrence and before sufficient time had elapsed for such new wounds to significantly increase in size or become chronic. The benefit of repeated dosing to treat wounds as early as possible after they occur is supported by the proportions of wounds that had healed at week 12: while 65% (109/168) of baseline wounds healed over 12 weeks, an equal proportion of 65% (50/77) of day-17 wounds healed from day 17 to week 12 (i.e., in only 9.5 weeks) and even 75% of day-35 wounds healed from day 35 to week 12 (in only 7 weeks). This suggests that the closer a wound gets to becoming chronic (i.e., lasting 12 weeks or more), the harder and longer it will take to heal.

Additionally, the herein observed delay in the development of new wounds (i.e., 77 wounds/17 days, 48 wounds/18 days, and 49 wounds/49 days for day-17, day-35, and week-12 wounds, respectively) (Figure 2) under a regime of repeated ABCB5^+^ MSC treatments was noteworthy. Crucial prerequisites for successful local anti-inflammatory and repair-promoting effects by systemically administered cells are efficient recruitment, migration, and homing of therapeutically grafted cells in response to chemokine gradients released from sites of injury [28,29]. In this regard, for the herein studied skin-derived human ABCB5^+^ MSC populations, in-vivo skin homing and engraftment capabilities have already previously been demonstrated in pre-clinical studies in recipient NSG mice: Systemically grafted human ABCB5^+^ MSCs homed to skin wounds and were detectable for at least 14 days, demonstrating a superior engraftment potential compared to side-by-side evaluated bone-marrow-derived MSCs [30]. Moreover, ABCB5^+^ MSC engraftment into uninjured skin has also been demonstrated in mice: Intravenously infused mouse ABCB5^+^ MSCs homed to the skin and survived for at least 17 days against a fully allogeneic barrier (BALB/c ABCB5^+^ MSCs grafted into C57/BL6 mice) [12]. These findings, together with the consideration that the permanent, intrinsic inflammatory environment in RDEB skin is associated with increased expression of genes related to immune system activation [31], increased neutrophil and CD38^+^ (M1) pro-inflammatory macrophage infiltration with high MHC II expression, and defective macrophage phenotype switching [32], potentially explain why RDEB skin might be prone to preferentially recruit systemically administered ABCB5^+^ MSCs to skin sites even before open wound manifestation, and hence our current clinical observation of inhibitory effects on new wound formation by systemically grafted allogeneic ABCB5^+^ MSCs.

**Figure 3:**
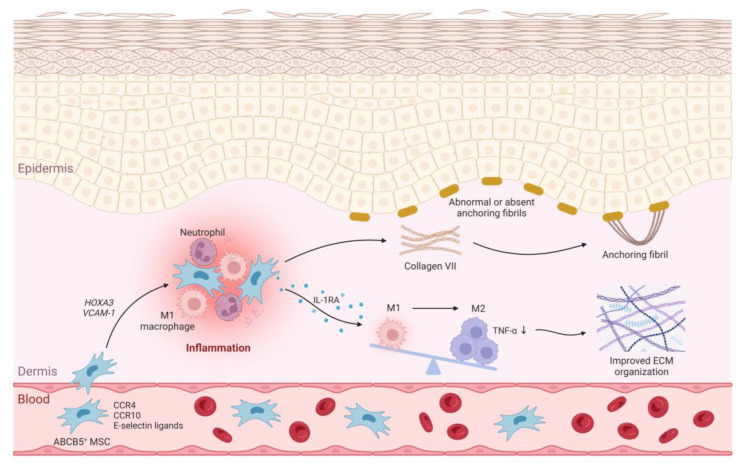
Hypothetical mechanisms of ABCB5^+^ MSCs to reduce inflammation and enhance stability in RDEB skin. Intravenously administered ABCB5^+^ MSCs migrate and home to the inflamed skin to induce a shift from pro-inflammatory M1 macrophages to pro-regenerative M2 macrophages. The resulting suppression of TNF-α signaling is expected to improve extracellular matrix organization. In addition, ABCB5^+^ MSCs can secrete collagen VII, the major component of the anchoring fibrils that attach the epidermis to the dermis within the basement membrane zone, which might contribute to further improving the structural integrity of the skin. ECM, extracellular matrix; IL-1RA, interleukin-1 receptor antagonist; M1, M1 macrophage; M2, M2 macrophage, TNF-α, tumor necrosis factor-alpha. Created with BioRender.com.

This possibility is further supported by previous findings that ABCB5^+^ MSCs possess the potential to ameliorate neutrophil overactivation [33] and to abrogate M1 macrophage persistence while inducing transition to anti-inflammatory, healing-promoting M2 macrophages [13]. Interestingly, M2 (CD206^+^) macrophage polarization in skin samples was found to be associated with improved wound healing and symptom relief in RDEB patients following three intravenous infusions of human UCB-MSCs [11]. It is noteworthy in this context that ABCB5^+^ MSCs-mediated induction of M2 macrophage phenotype switching has been shown to be associated with a significant reduction of tumor necrosis factor-alpha (TNF-α) signaling in the skin [13], while conversely, TNF-α is upregulated and pathophysiologically involved in RDEB [34,35,36]. Therefore, the current observations raise the possibility that systemically grafted ABCB5^+^ MSCs, beyond their known wound healing-promoting effects on established wounds [15,17,20], are also recruited to RDEB inflamed skin prior to wound formation and at such sites alleviate inherent M1 macrophage-driven inflammation, with concomitant improvements in extracellular matrix organization and tissue stabilization, and enhancement of damage resistance of RDEB skin [32] (Figure 3).

Finally, ABCB5^+^ MSCs are also capable of secreting type VII collagen [30], which raises the possibility that repeatedly grafted allogeneic ABCB5^+^ MSCs could additionally contribute to skin integrity improvements also through the provision of functional type VII collagen, lack of which is the primary cause of skin fragility in RDEB. In healthy skin, collagen VII trimerizes to form anchoring fibrils that connect the epidermal basement membrane with the dermal extracellular matrix [37] (Figure 3). In a collagen VII-hypomorphic mouse model of RDEB [38], intradermally injected human bone marrow-derived MSCs were capable of depositing collagen VII at the dermal-epidermal junction and enhancing skin resistance to shear forces through the de-novo formation of immature anchoring fibrils [39]. Intravenous MSC administration, as performed in the present trial, would hereby spare patients the discomfort of multiple intradermal injections while at the same time allowing for systemic treatment effects. While it has been postulated that, at least for bone marrow-derived MSCs, intravenous administration might not deliver sufficient numbers of cells to the skin [39], it is noteworthy that skin-derived ABCB5^+^ MSCs home in greater numbers to the skin upon intravenous infusion and possess superior collagen VII secretion capacity compared to bone marrow-derived MSCs [30]. Notably, increased collagen VII expression at the dermo-epidermal junction observed in an RDEB patient receiving three intravenous infusions of UCB-MSCs [11] supports the possibility of collagen VII delivery by systemic MSC treatment. Thus, further studies are warranted to determine whether intravenously infused ABCB5^+^ MSCs, at currently explored doses, are indeed capable of replacing defective collagen VII in RDEB skin at rates that could potentially confer therapeutic benefits.

## 5. Conclusions

Given the post-hoc nature of this analysis, the use of documentary, non-standardized photographs, and the lack of a control group, the findings reported herein should be considered hypothesis-generating, requiring further demonstration of validity in subsequent placebo-controlled trials. Under this premise, the identified therapeutic effects, which manifested themselves in decelerated RDEB cutaneous wound formation rates and accelerated and more stable healing of newly developed RDEB wounds, suggest a therapeutic advantage of repeated dosing of systemically administered ABCB5^+^ MSCs to patients with RDEB. Benefits were observed after both the first repeat infusion (18 days after the first infusion) and the second repeat infusion (7 weeks after the second infusion), which appears to support a strategy that combines induction therapy (with shorter intervals between infusions) with subsequent maintenance therapy (with longer intervals between infusions). In conclusion, this analysis suggests a systemic healing-promoting and skin-stabilizing effect of treatment with allogeneic ABCB5^+^ MSCs and provides valuable information for the identification of optimal dosing schemes as a further step on the way to efficient treatment strategies to respond to the urgent needs of patients suffering from RDEB.

## Figures and Tables

**Table 1:** Healing parameters of the new wounds that occurred on day 17 (“day-17 new wounds”) or day 35 (“day-35 new wounds) as compared to the wounds that were present at baseline.

Parameter		Day-17 New Wounds N = 77	Day-35 New Wounds N = 48	Baseline Wounds ^1^ N = 168
Healing ratio within:	17 days			45/168 (27%)
	18 days	43/77 (56%)		
	35 days			
	7 weeks		36/48 (75%)	
	9.5 weeks			
	12 weeks			109/168 (65%)
Median time to first wound closure		18 days ^2^		35 days
The proportion of durably (≥7 weeks) healed wounds ^3^		38/43 ^4^ (88%)		69/93 ^5^ (74%)

^1^ The results for the baseline wounds have been published previously [20]. ^2^ Inferred from the observation that >50% of the day-17 new wounds healed within 18 days. ^3^ Refers to the wounds that have remained closed for significantly longer than the typical time to recurrence, which natural history studies of RDEB have shown to be an average of 3 weeks [24]. ^4^ Due to the trial visit schedule, the proportion of durably closed wounds could only be determined for wounds that were closed by day 35 (n = 43). ^5^ Due to the trial visit schedule, the proportion of durably closed wounds could only be determined for wounds that were closed by day 17 and/or day 35 (n = 93).

## Data Availability

The data presented in this study are available on request from the corresponding author. The data are not publicly available due to privacy/ethical restrictions.
